# Virginia Medical Monthly

**Published:** 1874-06

**Authors:** 


We have received the April and May numbers of a new candidate for professional patronage, the "Virginia Medical Monthly." Landon B. Edwards, M.D., Editor and Proprietor. It is published at Richmond, at $2.00 per annum. The "Monthly" is highly creditable to its editor and proprietor, and deserves a good support from the profession it serves. We heartily welcome Dr. Edwards to the fraternity, and wish him the best encouragement in his new enterprise.

